# Argenti Nitras as a Remedy in the Treatment of Pyorrhœa

**Published:** 1892-09

**Authors:** E. H. Stebbins

**Affiliations:** Shelburne Falls, Mass.


### ARGENTI NITRAS AS A REMEDY IN THE TREATMENT OF PYORRHŒA.[1]

BY DR. E. H. STEBBINS, SHELBURNE FALLS, MASS.

THE value of remedial agents depends largely upon their continued good effects. Agents that are readily diluted or wasted by the fluids of the mouth are necessarily evanescent in their effects, and must be frequently applied in severe cases. No one remedy is best in all conditions. A splint that is sufficient to hold in place an infant's broken arm is wholly inadequate for a man's broken leg.

It is not the purpose of this paper to discuss remedies for incipient or mild forms of pyorrhœa, but those cases where other remedies have been ineffectual, and the disease has progressed till hope is nearly or entirely gone.

We recollect the late Dr. Riggs, of Hartford, and his treatment of pyorrhœa. His work in this line had a very good effect upon the profession in calling attention to the necessity of removing all and every particle of calcareous matter from the roots of teeth. Doubtless all of us can recall cases treated by him, or others, where the operations were performed as thoroughly as it seemed possible, and yet the deposit and accompanying pyorrhœa returned, and the same process must often be repeated.

How many patients have we to-day whose gums give us and them more annoyance and solicitude than their teeth do? How exasperating to our pride when, in spite of all our efforts, we are obliged to let a highly-esteemed patient lose one after another of his or her teeth!

It is not claimed that argenti nitras will cure *all* cases. It will not raise the dead. The long-continued good results of this agent in treatment of pyorrhœa of the gums is probably due to the compound formed by it with the organic substance of the cementum. This product being a powerful germicide, and held in close proximity to the diseased portion, is a perpetual and deadly foe to the microbes.

For the effects of the salts upon tooth-substances reference may be made to my paper published in the INTERNATIONAL DENTAL JOURNAL, October, 1891, and in the *Ohio Journal of Dental Science*, March, 1892.

The first effect of an application of the salts to purulent gums

---

[1] Read at a meeting of the New York Odontological Society, May 17, 1892.

is to turn them to a whitish color. The cauterized portion will slough in a day or two, leaving the remainder in a healthy condition. Cases where a slight pressure against the gums produced copious discharges of pus about the teeth have been treated once, and in a few days the whole appearance of the parts was found to have been changed, and to sustain hard pressure without causing any discharge of pus. Instead of the soft, fluctuating condition of the gingival border, the gums have presented a hard and apparently healthy condition. Where the teeth are very loose they become more firm in their sockets. Patients express with much satisfaction their appreciation of the results, and say their teeth and gums "feel so much better."

How long do the results last?

Case III., page 666, INTERNATIONAL DENTAL JOURNAL, October, 1891, has been under my observation since the first treatment. It is safe to say that since then the calcareous deposit has not been five per cent. of what it was before. Occasionally it has been necessary to retouch some places where the black crust has been worn off by his artificial denture, or some other cause. Recently gingivitis appeared by three teeth in different parts of the mouth. Upon pressure pus exuded. Some tartar was removed from deep down on the roots of the teeth, and the salts applied. Pyorrhœa and inflammation soon ceased.

Case IV., page 667, INTERNATIONAL DENTAL JOURNAL, has recently had tartar begin to deposit on the inferior incisors and the gum by them to be inflamed. This case has been over four years without any treatment. Other parts of the gums aside from that just mentioned are in very good condition, and there has been no deposit of tartar compared with what there was before treatment.

Other cases might be related, but they would only confirm those given. No case has come under my observation that did not yield gratifying results.

MANNER OF TREATMENT.

Remove all calcareous deposits from the teeth. (If any one has not extracted a tooth immediately after cleaning it, let him do so, and see how ineffectually the tartar has been removed.) With a slender wood point carry small quantities of the *pulverized* salts down beside the roots of the teeth till every exposed part thereof is touched as well as every portion of diseased gum. Make the application to but a few teeth at a time, so as to be able to keep the spreading of the dissolved salts under control. Keep the mouth as free from saliva as convenient. As soon as a few teeth have been treated inject water freely to carry away the surplus dissolved salts. Then proceed with a few more in the same manner until all are treated. Of course, care should be exercised to protect all other parts of the mouth. Care and skill should enable any operator to avoid touching the salts on the patient, or himself, where they are not needed. The use of paper napkins or pieces of cloth to wipe the patient's mouth will save staining other napkins.

It is a source of regret to me that it is impracticable to have a patient here to illustrate the subject under consideration. Inasmuch as there are patients present to show results of treatment for decay, I will give an account of a case which is of peculiar interest to me.

A young lady (not vigorous in health or constitution) consulted me in 1885 about the feasibility of saving her teeth. Some of them were filled, others were decayed. She had been advised to have nothing done till it should be necessary to have them all extracted. She was in a dilemma, but accepted my counsel and kept her teeth. All but seven of them have been filled. In April, 1886, I discovered quite a groove of decay in the central incisors, extending from the angle of labial and mesial surfaces across the mesial surfaces and around to near the middle of the palatal surfaces. The gum nearly covered these grooves. I applied the salts at once. In January, 1889, the left one had begun to decay a little farther along on the palatal surface, and was retouched with the salts. No other symptom of decay has appeared,—more than six years.

A little girl has just been to my office whose teeth I have treated. Over two years ago the distal surfaces of her first temporary molars and the mesial of the second ones were decayed. To save them till time for them to be replaced by the bicuspids it was necessary to fill or treat them with the nitrate. The latter was done, and they have been preserved, and will be till it is time to extract them, and that without filling. She has had other teeth treated that have resulted as favorably. The little boy of the same family has been as fortunate in this respect as the girl. The parents and children are very much pleased by the results.

My observations confirm my confidence in the efficacy of this agent. Sometimes I have treated large cavities with the salts, and at a subsequent sitting filled them with gutta-percha or cement, without excavating.

Are there objections? Yes.

A lady said to me, "If folks only knew what it is, so they would not think it is dirty decay, it would not be objectionable." Divest the minds of Americans of prejudice, and a clean, healthy, dark spot on a tooth would be preferable to a patch of gold. Much credit is due practitioners who inveigh against such glittering displays of the shining metal in the mouths of our people. But there are thousands of cavities out of sight that can be just as well saved by this method as by filling. It so completely fills the requirements for some agents that are adapted to preserve the teeth of children, —little children, nervous, timid children,—that it commends itself to the careful consideration of every dentist.

There is no doubt (in my mind) but more than three-fourths—yes, ninety per cent.—of children's teeth that decay can be preserved their allotted time of life if the treatment is begun in season and faithfully followed up. If we charge for value of services rendered, why not get better fees for this than for filling, considering the time it takes? Who would not use nitrate of silver, if by any plausible means a fee of five or ten dollars could be charged for each cavity? I am prompted to say this by the questions so often asked me about fees for it.

When I observe incipient decay in the teeth of a patient I recommend nitrate of silver to arrest its further progress. My advice is usually accepted, and the results are in most instances very satisfactory. Many patients freely express their approval of the treatment. Many times patients ask me what it does. My reply is that "it kills, embalms, and entombs the microbes just where it finds them."

